# Dauer signalling pathway model for *Haemonchus contortus*

**DOI:** 10.1186/s13071-019-3419-6

**Published:** 2019-04-29

**Authors:** Guangxu Ma, Tao Wang, Pasi K. Korhonen, Andreas J. Stroehlein, Neil D. Young, Robin B. Gasser

**Affiliations:** Department of Veterinary Biosciences, Melbourne Veterinary School, The University of Melbourne, Parkville, VIC 3010 Australia

**Keywords:** *Haemonchus contortus*, Dauer signalling pathway, Transcriptomic analysis, Proteomic analysis, Phospho-proteomic analysis

## Abstract

**Background:**

Signalling pathways have been extensively investigated in the free-living nematode *Caenorhabditis elegans*, but very little is known about these pathways in parasitic nematodes. Here, we constructed a model for the dauer-associated signalling pathways in an economically highly significant parasitic worm, *Haemonchus contortus*.

**Methods:**

Guided by data and information available for *C. elegans*, we used extensive genomic and transcriptomic datasets to infer gene homologues in the dauer-associated pathways, explore developmental transcriptomic, proteomic and phosphoproteomic profiles in *H. contortus* and study selected molecular structures.

**Results:**

The canonical cyclic guanosine monophosphate (cGMP), transforming growth factor-β (TGF-β), insulin-like growth factor 1 (IGF-1) and steroid hormone signalling pathways of *H. contortus* were inferred to represent a total of 61 gene homologues. Compared with *C. elegans*, *H. contortus* has a reduced set of genes encoding insulin-like peptides, implying evolutionary and biological divergences between the parasitic and free-living nematodes. Similar transcription profiles were found for all gene homologues between the infective stage of *H. contortus* and dauer stage of *C. elegans*. High transcriptional levels for genes encoding G protein-coupled receptors (GPCRs), TGF-β, insulin-like ligands (e.g. *ins-1*, *ins-17* and *ins-18*) and transcriptional factors (e.g. *daf-16*) in the infective L3 stage of *H. contortus* were suggestive of critical functional roles in this stage. Conspicuous protein expression patterns and extensive phosphorylation of some components of these pathways suggested marked post-translational modifications also in the L3 stage. The high structural similarity in the DAF-12 ligand binding domain among nematodes indicated functional conservation in steroid (i.e. dafachronic acid) signalling linked to worm development.

**Conclusions:**

Taken together, this pathway model provides a basis to explore hypotheses regarding biological processes and regulatory mechanisms (*via* particular microRNAs, phosphorylation events and/or lipids) associated with the development of *H. contortus* and related nematodes as well as parasite-host cross talk, which could aid the discovery of new therapeutic targets.

**Electronic supplementary material:**

The online version of this article (10.1186/s13071-019-3419-6) contains supplementary material, which is available to authorized users.

## Background

Signal transduction is a process by which physical or chemical impulses are transmitted through a cell *via* a series of molecular events [1, 2]. Many signalling pathways involve the binding of molecules (called ligands) to receptors that initiate or activate processes within cells. Usually, ligand-binding leads to conformational alteration(s) in a receptor, which triggers a chain of biochemical events that regulate processes such as transcription and translation, post-translational or conformational alterations of proteins and/or the location of expression [3–5].

Signalling pathways have been extensively investigated in the free-living nematode *Caenorhabditis elegans* [6, 7], which is arguably the best-characterised and most tractable metazoan model organism [8, 9]. Particularly well studied are pathways governing/regulating growth, development, reproduction and lifespan [10–14], including those of the canonical “dauer signalling” complex [15–19]. Here, environmental signals are received by chemosensory neurons through the cyclic guanosine monophosphate (cGMP) signalling pathway [15], then transduced *via* the transforming growth factor-β (TGF-β) signalling and insulin/insulin-like growth factor 1 (IGF-1) signalling pathways in neuroendocrine tissues [17, 18], and converted to steroid hormone signals (e.g. dafachronic acids, DAs) to regulate cellular processes critical to development, reproduction and lifespan [10, 11, 19, 20]. Although homologous signalling pathways have been proposed or partially described for parasitic nematodes [21–26], knowledge is incomplete and functional information mostly lacking. Clearly, the availability of comprehensive genomic and transcriptomic data sets for nematodes of the order Strongylida (parasite.wormbase.org) [27–31], which belong to the same evolutionary clade as *C. elegans*, provides a unique opportunity and foundation to explore the dauer signalling pathway in socioeconomically important representatives of this order.

*Haemonchus contortus* (barber’s pole worm) is one such representative, arguably one of the most important gastrointestinal nematodes of ruminants worldwide [32]. This worm has a complex life-cycle from the egg to the adult stage, under variable environmental and host conditions [33]. Specifically, the third-stage larvae (L3s) of *H. contortus* survive in the environment and will not develop to the next stage until activated by cues in the gut of the host animal; similarly, the fourth-stage larvae (L4s) may arrest in development within the host in response to seasonal and/or host factors [34–36]. Although previous studies have explored aspects of TGF-β and IGF-1 signalling pathways in *H. contortus* and investigated the functionality of selected molecules, including *Hc-hsp-90*, *Hc-daf-16* and *Hc-daf-2* [22, 37–44], there are still significant gaps in our knowledge and understanding of the signalling pathways linked to dauer in this species.

Here, employing extensive, publicly available genomic and transcriptomic datasets for *H. contortus*, and guided by molecular and functional resources available for *C. elegans*, we infer the gene homologues representing four inter-connected (dauer-associated) signalling pathways in *H. contortus* and explore developmental transcriptomic, proteomic and phosphoproteomic profiles for pathway components. This pathway reconstruction and validation should provide a basis for functional studies of dauer signalling in *H. contortus* and related nematodes, and might facilitate the discovery of new anthelmintic targets.

## Methods

### Identifying *C. elegans* gene homologues in *H. contortus*

A list of all genes (*n* = 102) and gene products (*n* = 182) representing the cGMP, TGF-β and IGF-1 signalling pathways as well as the steroid hormone pathway in *C. elegans* was established based on published information [16, 39, 45] (Additional file 1: Table S1). The gene and protein sequences, their accession numbers and transcriptomic data were obtained from WormBase (v.WS261). Homologues of these genes were identified by searching (tblastn; *e*-value: ≤ 10^−5^) the *C. elegans* protein sequences against gene predictions from the latest, published genome and the transcriptomes of *H. contortus* [28–31]. The *C. elegans* protein sequences were also searched against the *H. contortus* genome using BLAT v.34 [46] to identify homologues. The gene sequences identified were compared (blastx; *e*-value: ≤ 10^−5^) with *C. elegans* proteins (PRJNA13758.WS261) to cross-verify their identity.

### Gene curation and structural modelling

Genes and transcripts were curated using a recently established method [47]. In brief, the sequences inferred to represent homologues were mapped to the genome assembly of *H. contortus* using the program BLAT v.34; mapping was displayed using the Integrated Genome Viewer v.2.4.4 (IGV). The mapped transcripts were reassembled using the program CAP3 [48] for possible extensions. The reassembled transcript sequences were mapped to the genome assembly of *H. contortus* [29], and the corresponding coding DNA sequences (CDS) in the genome were refined using the “coding2genome” model in the program Exonerate v.2.2.0 [49]. The sequences of curated genes were cross-checked with those of complementary DNAs (cDNAs) for *Hc-daf-16*, *Hc-daf-2* and *Hc-pdk-1* [41, 43, 44]. Subsequently, open reading frames (ORFs) were predicted using the program ORF finder [50], and structural and functional domains identified using InterProScan v.61.0 [51, 52]. Pairwise comparisons of inferred amino acid sequences were performed using the program MAFFT v.7.309 [53].

Structural modelling was conducted for a nuclear hormone receptor (DAF-12) using the program I-TASSER [54], following the alignment of amino acid sequence data in MAFFT v.7.309. The alignment was viewed in MView v.1.62 [55]. Models were displayed and compared with available crystal structures [56] using UCSF Chimera v.1.12 [57], and structural similarities between query and template sequences were measured using TM-score and root-mean-square deviation (RMSD) [54]. Biological functions (Gene Ontology, GO) of the modelled protein domain were inferred based on structural similarity.

### Transcription analysis

RNA-seq reads (paired-end) from individual developmental stages/sexes of the nematode were mapped to individual curated CDS in the genome using Bowtie v.2.1.0 within the software package RSEM v.1.2.11 [58, 59]. At least 10 reads needed to map to a CDS for transcription to be recorded. Transcription levels of messenger RNAs (mRNAs) were recorded in fragments per kilobase per million mapped reads (FPKM). For individual genes of individual developmental stages of *H. contortus*, transcription levels were displayed in a heat map using heatmap.2 in an R-language environment (v.3.5.1).

### Protein analyses

Proteomic analysis of *H. contortus* was conducted using an established protocol [31]. In brief, protein sequences predicted from individual homologous genes were used to search mass spectrometric (MS) data representing the egg, L3, L4 (female and male) and adult (female and male) stages of *H. contortus* using Proteome Discoverer software v.2.0 (Thermo Fisher Scientific, San Jose, CA, USA). Peptides were identified using a false discovery rate (FDR) cut-off of < 1% at the peptide and protein levels. Peptide intensities were calculated using Spectronaut software v.11 (Biognosys). At least two peptides needed to match a corresponding protein sequence for expression to be recorded. Peptide intensities were employed to infer the expression levels of individual protein homologues in different developmental stages of *H. contortus*. A phosphoproteomic analysis of egg, L3, L4 (female and male) and adult (female and male) stages of *H. contortus* was conducted using an established TiO_2_ enrichment protocol [60, 61]. Protein sequences encoded by dauer signalling gene homologues were employed to interrogate the phosphoproteomic data using the Proteome Discoverer software. Phosphopeptides were identified using a FDR cut-off of < 1% at the peptide and protein levels. Phosphorylated proteins were mapped to dauer signalling pathways in *H. contortus*.

## Results

### The dauer signalling pathway model

**Fig. 1:**
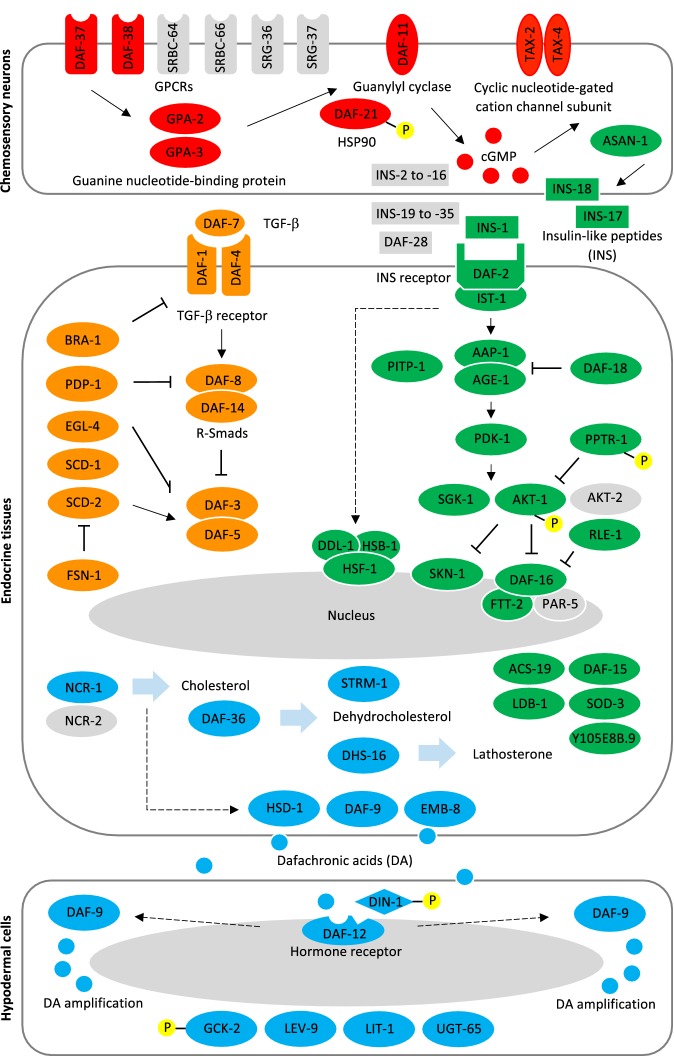
Dauer signalling pathway model proposed for *Haemonchus contortus*. The cGMP (red), TGF-β (orange), insulin-like growth factor 1 (IGF-1) (green) and steroid hormone (light blue) signalling pathways are constructed based on information and data available for *C. elegans*. Pathway components not identified in *H. contortus* are indicated in grey. It is hypothesized that environmental signals are received by G-protein-coupled receptors (GPCRs) through cGMP signalling in chemosensory neurons, transduced *via* TGF-β and insulin-like peptides in parallel TGF-β signalling and IGF-1 signalling, which then converge into the steroid hormone signalling pathway *via* the biosynthesis of dafachronic acids (DAs; ligands of nuclear hormone receptor DAF-12) in neuroendocrine tissues, determining the activation of DAF-12 in target (hypodermal) cells. The activation of DAF-12 initiates or regulates numerous molecular events such as transcription. Signalling or regulation is indicated by an arrow or cross-line. Phosphorylation events detected in the third larval stage (L3) of *H. contortus* are indicated by a phosphate group (yellow). Dashed arrows indicate pathways which need to be further elucidated. Drawing is adapted or modified from previous publications [24, 39, 45, 62]

First, we identified the genes (*n* = 102; Additional file 1: Table S1) representing the cGMP, TGF-β, IGF-1 and steroid hormone signalling pathways in *C. elegans*, and constructed the canonical framework for the dauer signalling pathway model (Fig 1).

Secondly, we identified all homologues of these genes in *H. contortus*. To do this, we conducted exhaustive homology searches against the genome and combined transcriptome of *H. contortus*, which yielded more than 25,000 sequences in *H. contortus*, from which we predicted genes and transcripts. These genes and transcripts, relating to 61 full-length coding sequences, were curated and assigned names (Additional file 1: Table S2). *Hc-daf-1* was represented by two paralogues (Additional file 1: Table S2). All 61 genes were transcribed, and transcripts had domain sequence-matches with respective amino acid sequences encoded by *C. elegans* genes (Additional file 1: Table S2).

Thirdly, we mapped the 61 genes to the framework constructed for *C. elegans* (Fig. 1), providing a model for dauer signalling pathways in *H. contortus*. From this mapping, 60 of the 102 *C. elegans* dauer signalling genes were homologous to the 61 genes in *H. contortus*. However, not detected in the parasitic nematode were genes encoding proteins SRBC-64, SRBC-66, SRG-36 and SRG-37 (G protein-coupled receptors, GPCRs); AKT-2 (serine/threonine kinase), PAR-5 (14-3-3 protein), DAF-28 (insulin), INS-2 to INS-16 and INS-19 to INS-37 (insulin-like peptides); and NCR-2 (membrane permease), inferred or known to be involved in dauer signalling in *C. elegans*.

### Signalling cascade

Based on our model for *H. contortus*, we hypothesise the following events in the four components of the signalling cascade:

For cGMP signalling (8 components; Additional file 1: Table S2), environmental cues are sensed by *Hc-*DAF-37 and *Hc*-DAF-38 (GPCRs) that activate *Hc-*GPA-2 and *Hc*-GPA-3 (GTPase), which initiates cGMP production by *Hc-*DAF-11 (receptor-like guanylyl cyclase) and *Hc-*DAF-21 (heat shock protein 90), controlling the activity of the transmembrane channels formed by *Hc-*TAX-2 and *Hc*-TAX-4 (cGMP-gated channel subunits).

For TGF-β signalling (14 components; Additional file 1: Table S2), *Hc-*DAF-7 (TGF-β) is bound by *Hc-*DAF-1 and *Hc*-DAF-4 (heterotetrameric TGF-β receptor), which activates *Hc*-DAF-8 and *Hc*-DAF-14 (Smad protein complex) that is inhibited by *Hc*-PDP-1 (serine/threonine phosphatase), inhibiting the function of *Hc-*DAF-3 and *Hc*-DAF-5 (transcription regulator complex) which are regulated by *Hc-*EGL-4 (cGMP-dependent protein kinase), *Hc*-SCD-1 and *Hc*-SCD-2 (receptor tyrosine kinases) and *Hc*-FSN-1 (F-box synaptic protein).

For IGF-1 signalling (26 components; Additional file 1: Table S2), *Hc*-ASNA-1 (ATPase) promotes the secretion of *Hc-*INS-1, *Hc*-INS-17 and *Hc*-INS-18 (insulin-like peptides) from chemosensory neurons, which bind to *Hc-*DAF-2 and *Hc*-IST-1 (receptor tyrosine kinase and substrate) that regulate sequential phosphorylation by *Hc-*AAP-1, *Hc*-AGE-1 (phosphoinositide 3-kinase), *Hc*-PITP-1 (phosphatidylinositol transfer protein), *Hc*-PDK-1 (3-phosphoinositide-dependent kinase), *Hc*-SGK-1 and *Hc*-AKT-1 (serine/threonine kinases), regulating the cytoplasmic sequestration of transcription factors *Hc-*HSF-1 (with *Hc-*DDL-1 and heat shock binding protein *Hc-*HSB-1), *Hc*-SKN-1 and *Hc*-DAF-16 (with 14-3-3 protein FTT-2). *Hc-*DAF-18 (lipid phosphatase), *Hc*-PPTR-1 (serine/threonine phosphatase) and *Hc*-RLE-1 (E3 ubiquitin ligase) regulate the phosphorylation of *Hc-*AGE-1, *Hc*-AKT-1 and *Hc*-DAF-16, respectively. The interactors in this signalling pathway also include *Hc-*ACS-19 (acyl-CoA synthetase), *Hc*-DAF-15 (raptor protein), *Hc*-LDB-1 (LIM Domain Binding protein), *Hc*-SOD-3 (iron/manganese superoxide dismutase) and a homologue of Y105E8B.9 (glutathione transferase).

For steroid hormone signalling (13 components; Additional file 1: Table S2), extracellular cholesterol and glycolipids are transported into neuroendocrine cells by *Hc-*NCR-1 (membrane permease), and sequentially modified by *Hc-*DAF-36 (Rieske oxygenase), *Hc*-STRM-1 (methyltransferase), *Hc*-DHS-16 (dehydrogenase), *Hc*-DAF-9 (cytochrome P450) and *Hc*-EMB-8 (NADPH-cytochrome P450 reductase), or directly modified by an *Hc-*HSD (hydroxysteroid dehydrogenase), resulting in the production of DAs that activate *Hc-*DAF-12. In turn, the activation of *Hc-*DAF-12 promotes the production of DAs *via Hc-*DAF-9 in target (hypodermal) cells. The binding of DA or *Hc-*DIN-1 (nucleic acid binding protein) to *Hc-*DAF-12 (nuclear hormone receptor) forms a co-activator or co-repressor complex, regulating downstream *Hc-*GCK-2 (mitogen-activated protein kinase kinase kinase kinase), *Hc*-LEV-9 (sushi domain-containing protein), *Hc*-LIT-1 (serine threonine protein kinase) and *Hc*-UGT-65 (UDP glucuronosyltransferase).

### Molecular profiles throughout development

**Fig. 2:**
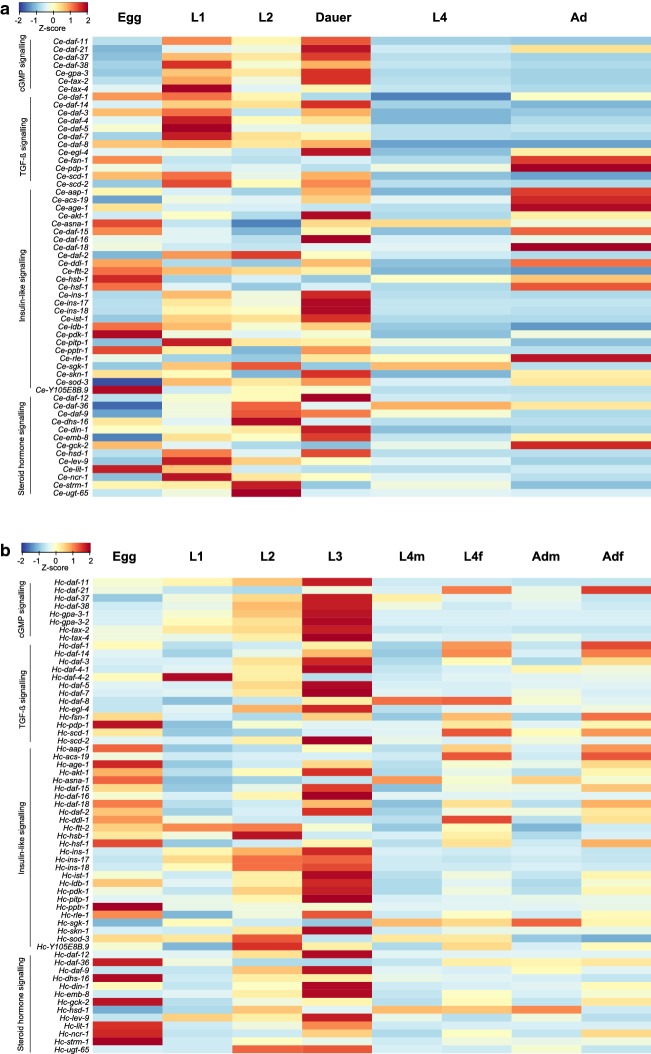
Transcription of dauer signalling genes in *Caenorhabditis elegans* and in *Haemonchus contortus*. Messenger RNA transcriptional levels (fragments per kilobase per million mapped reads, FPKM) of **a** 60 dauer signalling genes in key developmental stages of *C. elegans* [egg, the first- (L1), second- (L2), dauer-, fourth- (L4) stage larvae and adults (Ad)], and **b** all 61 homologous genes in *H. contortus* stages (egg, L1, L2, L3, L4 (female and male L4s—L4f and L4m), and female and male adults—Adf and Adm) are indicated in the heat maps. Colour scales and Z-scores indicate scaled FPKMs in the rows. Genes involved in the cGMP, TGF-β, IGF-1 and steroid hormone signalling pathways are listed

We established the transcription profiles of dauer signalling genes for all key developmental stages of *H. contortus* (egg, the first-larval (L1), second-larval (L2), L3, L4 stages, and female and male adult stages) and compared these profiles with those inferred for homologues in respective stages of *C. elegans*. In both nematode species, each homologous gene was transcribed in at least one developmental stage. Transcription profiles were similar between the L3 stage of *H. contortus* and dauer stage of *C. elegans* (Fig 2). In these developmental stages of the two species, peak transcription levels were detected for genes *daf-37*, *gpa-2*, *gpa-3, daf-11* and *tax-2* in the cGMP signalling pathway; *daf-14* and *egl-4* in the TGF-β signalling pathway; *ins-1*, *ins-17*, *ins-18*, *ist-1*, *akt-1*, *skn-1* and *daf-16* in the IGF-1 signalling pathway; and *emb-8*, *daf-12* and *din-1* in the steroid hormone signalling pathway (Fig. 2; Additional file 1: Tables S1 and S3). By contrast, there were distinct differences in profiles for other genes. Although high transcription levels were recorded for *daf-38* and *tax-4* (cGMP signalling), *daf-7*, *daf-1*, *daf-4*, *daf-3* and *daf-5* (TGF-β signalling), *daf-2* and *pitp-1* (IGF-1 signalling), and *daf-9* and *lev-9* (steroid hormone signalling) in *H. contortus* L3, transcriptional peaks for these genes were found in L1 or L2 stages of *C. elegans* (Fig. 2; Additional file 1: Tables S1 and S3). Marked differences in the transcription of selected genes, including *daf-21*, *daf-1*, *daf-4* and *daf-2*, were found between the two species in the egg, L4 and adult stages (Fig. 2).

We also explored the protein expression of the dauer signalling genes in *H. contortus* as well as the phosphorylation of proteins. In total, proteins encoded by 11 signalling genes were detected in the egg, L3, L4 and/or adult stages of the parasite (Additional file 1: Table S4). These genes included *Hc-daf-21* (cGMP signalling), *Hc-egl-4* and *Hc-pdp-1* (TGF-β signalling pathway), *Hc-akt-1*, *Hc-pptr*-*1*, *Hc-asna-1*, *Hc-ftt-2* and *Hc-sod-3* (IGF-1 signalling), and *Hc-emb-8*, *Hc-din-1* and *Hc-gck-2* (steroid hormone signalling). In these proteins, 1 to 6 phosphorylation sites was/were detected for *Hc*-DAF-21 (cGMP signalling), *Hc*-AKT-1 and *Hc*-PPTR-1 (IGF-1 signalling), *Hc*-DIN-1 and *Hc*-GCK-2 (steroid hormone signalling) (Additional file 1: Table S4). These five phosphorylated proteins were detected in egg and/or larval stages of *H. contortus*, particularly L3. However, none of them was found to be phosphorylated in female or male adult stages of this species.

### DAF-12—the checkpoint in dauer signalling

**Fig. 3:**
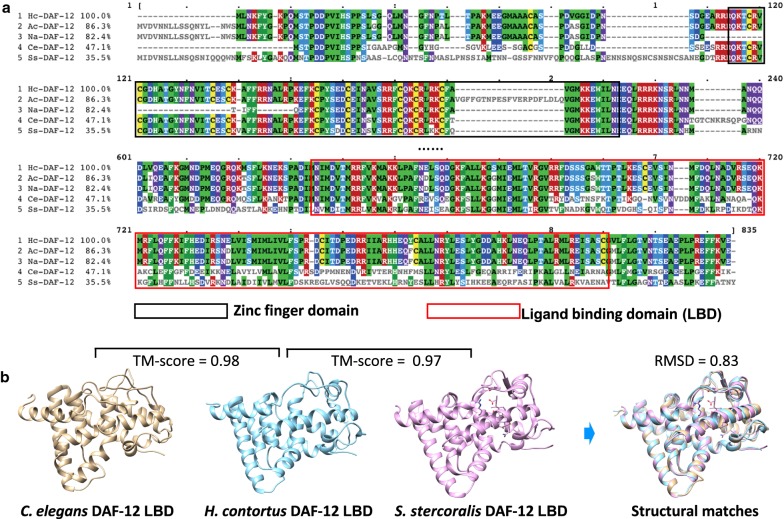
DAF-12 sequence and structural similarities among nematodes. **a** Alignment of the inferred protein sequences of DAF-12 of *Haemonchus contortus* (*Hc*-DAF-12), *Ancylostoma ceylanicum* (*Ac*-DAF-12; GenBank: EPB79655), *Necator americanus* (*Na*-DAF-12; GenBank: XP_013307217), *Caenorhabditis elegans* (*Ce*-DAF-12; WormBase ID: CE27585) and *Strongyloides stercoralis* (*Ss*-DAF-12; GenBank: AAD37372) is shown, with sequence similarities indicated. Zinc finger domain and ligand binding domain (LBD) are framed in black and red, respectively. **b** Structure models of LBDs of *H. contortus* and *C. elegans* are matched to the published crystal structure (PDB accession no. 3GYU_ChainA; *S. stercoralis*); TM-scores (0.98 and 0.97) and an overall root-mean-square deviation (RMSD) of 0.83 indicate high pairwise structural similarities

As DAF-12 is a determining point in the dauer signalling pathway, we explored its features and conservation. Pairwise comparisons of the inferred DAF-12 protein sequences of *H. contortus* (*Hc-*DAF-12a), *C. elegans* (WormBase ID: CE27585), *Strongyloides stercoralis* (GenBank: AAD37372), *Ancylostoma ceylanicum* (GenBank: EPB79655) and *Necator americanus* (GenBank: XP_013307217) revealed sequence identities of 35.5% to 86.3%. High sequence similarities were in the zinc finger (nuclear hormone receptor type) and ligand binding domains (Fig. 3a). The three-dimensional models for the DAF-12 ligand binding domains of *H. contortus* (3.1 ± 2.2 Å) and *C. elegans* (3.1 ± 2.2 Å) both showed high structural similarity (TM-score: ≥ 0.97) to the published crystal structure for DAF-12 (ligand-binding domain 3GYU_chainA; *S. stercoralis*) (Fig 3b). Based on sequence and structural similarities, the biological processes for *Hc-*DAF-12 were predicted as DNA-templated regulation of transcription (GO:0006355), steroid hormone-mediated signalling pathway (GO:0043401) and ecdysone receptor-mediated signalling pathway (GO:0035076), with GO-scores of 0.94, 0.94 and 0.67, respectively.

## Discussion

Using the enhanced genome and comprehensive transcriptome data sets for *H. contortus* as well as extensive information and data sets available for the related free-living nematode, *C. elegans*, we constructed a model for all four dauer signalling pathway components in *H. contortus* (Fig. 1). Based on this model, we explored gene transcription, expression and phosphorylation of the signalling molecules in all key developmental stages of *H. contortus*, compared the transcriptional profiles with those of stages in *C. elegans*, and examined the structural and likely functional similarities in a key determining point of the dauer signalling pathway, DAF-12, between these two nematodes. We believe that our model provides a basis to test hypotheses regarding the functions of these signalling pathway components in *H. contortus*, to elucidate the developmental biology of this and related nematodes, and to facilitate the discovery of new anthelmintic targets.

The identification of homologous GPCRs, ligands and receptors, kinases and phosphatases, transcriptional factors and a nuclear hormone receptor (DAF-12) in *H. contortus* implies relative conservation of dauer signalling, a statement which is, to some extent, supported by functional data for genes including *age-1*, *aap-1*, *daf-2*, *daf-7*, *daf-21*, *daf-16* and *pdk-1* in *H. contortus* [40–44, 63]. Specifically, previous genetic complementation studies have shown that *Hc-daf-2*, *Hc-daf-16* and *Hc-daf-21* could at least partially rescue respective null mutants in *C. elegans* [40, 41, 43], indicating functionality in the regulation of developmental processes in *H. contortus*.

Although similarities in dauer signalling have been proposed [21–25, 64] and verified through *in silico* studies [39, 45], there are some differences in the number of signalling homologues between *C. elegans* and parasitic nematodes. In the present study, although genes coding for pheromone receptors (*daf-37* and *daf-38*) and insulin-like peptides (i.e. *ins-1*, *ins-17* and *ins-18*) that promote dauer formation [18, 60–67] were identified in *H. contortus*, homologues coding for insulin-like peptides (e.g. *daf*-28, *ins-4* and *ins-6*) that promote larval development [18, 68] were not detected. Such variation in the number of genes encoding signalling molecules (particular GPCRs, GTPase, insulin-like peptides, serine/threonine kinase and membrane permease) has been reported in previous studies of parasitic nematodes including *Ascaris suum*, *N. americanus* and *S. stercoralis* [39, 45]. For example, *S. stercoralis* has only a small number of genes encoding insulin-like peptides, but has an expanded set of TGF-β-coding genes [45]. Although gene predictions depend significantly on the quality of the draft genomes used and bioinformatic algorithms applied [69, 70], the differences seen here are likely explained by the divergent evolution of nematodes, reflecting the marked biological differences among nematodes, both free-living and parasitic [14, 24, 26, 39]. Understanding the impact of evolutionary and biological divergences requires detailed studies of a broader range of nematodes species and functional assessments of particular dauer signalling components in them.

The dauer signalling pathway in *H. contortus* appears to play a role in larval diapause. The similar transcriptional patterns of signalling molecules between the infective L3 stage of *H. contortus* and the dauer stage of *C. elegans* indicate a similar function. The similarity in the arrested developmental state between the infective larva (L3) of strongylid nematodes and the dauer form in *C. elegans* has been discussed extensively [21, 22, 71], and the hypobiotic stage was proposed to be a pre-adaptation to parasitism (i.e. dauer hypothesis) [24], although we hasten to add that the L4 stage of some species of parasitic nematodes can undergo hypobiosis in host tissues and seems to be commensurate with dauer [35, 72–74]. This dauer hypothesis is supported by transcriptomic and proteomic evidence from the present study. Specifically, the transcriptional peaks of *daf-37* and *daf-38, daf-7*, *ins-1*, *ins-17* and *ins-18*, and *daf-16* in the L3 stage suggest a role in developmental arrest, whereas no translation and high transcription of genes *daf-9*, *daf-12* and *din-1* imply an adaptation (e.g. pre-transcription and storage of RNA) for the subsequent parasitic stage of *H. contortus*. Interestingly, the transcription of *daf-7* peaks in L3s of *H. contortus* (clade V) and of parasitic nematodes representing other clades, including *S. stercoralis* (clade IV) and *Brugia malayi* (clade III); the transcription profiles in these nematodes is distinct from the free-living nematode *C. elegans* (clade V), suggesting a “repurposing” of roles from signalling to other biological processes, such as immunomodulation, in parasitic nematodes [24, 25].

The roles of GPCRs, TGF-β, insulin-like ligands and fork head transcription factor (i.e. DAF-16) in dauer formation, and cytochrome P450 and nuclear hormone receptor (i.e. DAF-12) in regulating dauer formation in *C. elegans* have been reported previously [10, 65–67, 75]. However, there is a dearth of information on the functional roles of dauer signalling pathway components in developmental diapause of *H. contortus* [37]. Some explorations of parasitic nematodes, including *A. caninum* and *S. stercoralis* [76–79], provide functional evidence for cGMP and IGF-1 signalling, and particularly for steroid hormone (DA) signalling, in regulating the activation of infective larvae. Other studies have suggested roles of signalling pathways in hypobiosis (at the L4 stage) in *H. contortus* [37, 80] and related nematodes including *Ostertagia ostertagi*, *Teladorsagia circumcincta* and *Dictyocaulus viviparus* [34, 74, 81–84]. Moreover, the different transcriptional profiles of signalling genes in L4 and/or adult stages between *H. contortus* and *C. elegans* might relate to variation in sexual differentiation between these species. Divergent transcription profiles of signalling molecules have also been reported for nematodes such as *Heterodera glycines*, *Heligmosomoides polygyrus* and *S. stercoralis* [45, 63, 85], suggesting biological divergence in larval diapause and reproductive development among nematode species. For example, two orphan genes *Hc-daf-22* and *Hc-fau* have been inferred to regulate larval diapause in *H. contortus* [86, 87]. Clearly, functional analyses of signalling cascade components and their regulation are required to improve our understanding of molecular pathways governing developmental processes in parasitic nematodes.

The structural and functional similarities of DAF-12 among free-living and parasitic nematodes suggest a common theme (i.e. a hormone-signalling module) in developmental regulation [64, 88]. Specifically, the central roles of steroids in the development of nematodes have been extensively reported and discussed, particularly the roles of DA-DAF-12 in regulating a connected network of genes [19, 20, 62, 89–91]. Therefore, as a central point in the dauer signalling pathway, DAF-12 has been proposed as a therapeutic target in parasitic nematodes [56, 92, 93]. In addition, non-coding small RNAs might play roles in regulating signalling pathways in *H. contortus* [31]. For example, although substantial transcription of *Hc-daf-9* and *Hc-daf-12* (i.e. genes involved in promoting reproductive development) [10, 94] was detected in the L3 stage of *H. contortus*, the functions of these genes might be suppressed based on evidence of limited gene translation in this stage. In addition, the marked discrepancies in transcription and expression for genes such as *Hc-daf-21*, *Hc-daf-7* and *Hc-emb-8* might be explained by post-transcriptional regulation through microRNAs (miRNAs) [95, 96], as tight regulation by such RNAs has been inferred recently for *H. contortus* [31]. Nonetheless, our understanding of the biological roles of miRNAs in *H. contortus* is in its infancy [31, 97], which contrasts the situation for *C. elegans* where the regulatory roles of miRNAs in development are well studied [98, 99]. For instance, it has been reported that a complex feedback circuit between *daf-12* and the *let-7* family miRNAs regulates the division of epidermal stem cells during larval development [13, 75].

In addition to post-transcriptional regulation, post-translational modifications are also likely involved in dauer signalling in *H. contortus*. This statement is supported by the present phosphoproteomic results. Phosphorylation was detected on *Hc*-DAF-21, *Hc*-PPTR-1, *Hc*-AKT-1, *Hc*-DIN-1 and *Hc*-GCK-2 in the L3 stage of *H. contortus*, indicating an activation of cGMP, insulin-like and steroid hormone signalling pathways. Specifically, the exclusive phosphorylation of *Hc*-DAF-21, *Hc*-DIN-1 and *Hc*-GCK-2 in L3s suggests roles in arrested development. For *C. elegans*, it has been reported that phosphorylated PPTR-1 might activate the function of DAF-16 by reducing the inhibition of AKT-1, and that phosphorylated DIN-1 can inhibit the function of DAF-12 by forming a repression complex, promoting dauer diapause in this free-living nematode [12, 18]. The identification of a range of kinases and their functional assessment in *H. contortus* also indicate extensive phosphorylation during signal transduction and integration and encourage future explorations of nematode tissues to better understand the roles of miRNAs, phosphorylation and lipids in signal transduction events and to discover novel anthelmintic targets. Clearly, available genetic technologies, ‘omic resources, informatic tools and hypotheses should facilitate such investigations in *H. contortus* and related parasitic worms.

## Conclusions

Here, we propose a model for the canonical (dauer-associated) cGMP, TGF-β, IGF-1 and steroid hormone signalling pathways from genomic and transcriptomic data sets for *H. contortus*, guided by resources and data available for *C. elegans*. This model should provide a basis to test hypotheses pertaining to these pathways and a platform for future functional explorations of molecular biological processes and associated regulatory mechanisms (*via* miRNAs, phosphorylations and/or lipids) involved in the development and reproduction of *H. contortus* and related nematodes as well as parasite-host cross-talk, which might guide the discovery of novel therapeutic targets. Although the focus of the present paper was on *H. contortus*, the approach employed for pathway reconstruction could be applied to other nematodes that are relatively closely related to *C. elegans*, including, for instance, *Ostertagia* and *Trichostrongylus* (clade V), and *Ascaris* and *Toxocara* (clade III).

## Additional file

**Additional file 1: Table S1.** Salient information and data for genes known or inferred to be involved in the dauer signalling pathway in *Caenorhabditis elegans*. **Table S2.** Dauer signalling gene homologues in *Haemonchus contortus.* **Table S3.** Gene transcription of dauer signalling gene homologues in key developmental stages of *Haemonchus contortus.* **Table S4.** Protein expression and phosphorylation of dauer signalling gene homologues in key developmental stages of *Haemonchus contortus*.

## Abbreviations

cGMP: chemosensory neurons through the cyclic guanosine monophosphate
TGF-β: transforming growth factor-β
IGF-1: insulin/insulin-like growth factor 1
DAs: dafachronic acids
MS: mass spectrometry
